# Epigenetic Regulation of a Murine Retrotransposon by a Dual Histone Modification Mark

**DOI:** 10.1371/journal.pgen.1000927

**Published:** 2010-04-29

**Authors:** Reinhard Brunmeir, Sabine Lagger, Elisabeth Simboeck, Anna Sawicka, Gerda Egger, Astrid Hagelkruys, Yu Zhang, Patrick Matthias, Wolfgang J. Miller, Christian Seiser

**Affiliations:** 1 Max F. Perutz Laboratories, Medical University of Vienna, Vienna Biocenter, Vienna, Austria; 2 Department of Clinical Pathology, Medical University of Vienna, Vienna, Austria; 3 Friedrich Miescher Institute for Biomedical Research, Novartis Research Foundation, Basel, Switzerland; 4 Laboratories of Genome Dynamics, Center of Anatomy and Cell Biology, Medical University of Vienna, Vienna, Austria; University of Liège, Belgium

## Abstract

Large fractions of eukaryotic genomes contain repetitive sequences of which the vast majority is derived from transposable elements (TEs). In order to inactivate those potentially harmful elements, host organisms silence TEs via methylation of transposon DNA and packaging into chromatin associated with repressive histone marks. The contribution of individual histone modifications in this process is not completely resolved. Therefore, we aimed to define the role of reversible histone acetylation, a modification commonly associated with transcriptional activity, in transcriptional regulation of murine TEs. We surveyed histone acetylation patterns and expression levels of ten different murine TEs in mouse fibroblasts with altered histone acetylation levels, which was achieved via chemical HDAC inhibition with trichostatin A (TSA), or genetic inactivation of the major deacetylase HDAC1. We found that one LTR retrotransposon family encompassing virus-like 30S elements (VL30) showed significant histone H3 hyperacetylation and strong transcriptional activation in response to TSA treatment. Analysis of VL30 transcripts revealed that increased VL30 transcription is due to enhanced expression of a limited number of genomic elements, with one locus being particularly responsive to HDAC inhibition. Importantly, transcriptional induction of VL30 was entirely dependent on the activation of MAP kinase pathways, resulting in serine 10 phosphorylation at histone H3. Stimulation of MAP kinase cascades together with HDAC inhibition led to simultaneous phosphorylation and acetylation (phosphoacetylation) of histone H3 at the VL30 regulatory region. The presence of the phosphoacetylation mark at VL30 LTRs was linked with full transcriptional activation of the mobile element. Our data indicate that the activity of different TEs is controlled by distinct chromatin modifications. We show that activation of a specific mobile element is linked to a dual epigenetic mark and propose a model whereby phosphoacetylation of histone H3 is crucial for full transcriptional activation of VL30 elements.

## Author Summary

The majority of genomic sequences in higher eukaryotes do not contain protein coding genes. Large fractions are covered by repetitive sequences, many of which are derived from transposable elements (TEs). These selfish genes, only containing sequences necessary for self-propagation, can multiply and change their location within the genome, threatening host genome integrity and provoking mutational bursts. Therefore host organisms have evolved a diverse repertoire of defence mechanisms to counteract and silence these genomic parasites. One way is to package DNA sequences containing TEs into transcriptionally inert heterochromatin, which is partly achieved via chemical modification of the packaging proteins associated with DNA, the histones. To better understand the contribution of histone acetylation in the activation of TEs, we treated mouse fibroblasts with a specific histone deacetylase inhibitor. By monitoring the expression of ten different types of murine mobile elements, we identified a defined subset of VL30 transposons specifically reactivated upon increased histone acetylation. Importantly, phosphorylation of histone H3, a modification that is triggered by stress, is required for acetylation-dependent activation of VL30 elements. We present a model where concomitant histone phosphorylation and acetylation cooperate in the transcriptional induction of VL30 elements.

## Introduction

Our present view on transcriptional regulation has substantially advanced in recent decades. The classical model, that the presence of promoter sequences and the availability of transcription factors determine the expression status of corresponding genes, has been extended to a model, in which the accessibility of the DNA is central to transcriptional control. In eukaryotes, DNA is packed and compacted into chromatin with the nucleosome consisting of DNA and histone proteins as the basic unit. The degree of compaction – either into inaccessible heterochromatin or open euchromatin – has major implications for the transcriptional potential of associated DNA. A way to regulate chromatin accessibility is the posttranslational chemical modification of histone proteins. It can alter chromatin structure and switch genes from a transcriptional repressed to an active state and *vice versa* [1].

The best-studied histone modification is the acetylation of N-terminal lysine residues, which correlates with open chromatin and active gene transcription. In contrast, histone deacetylation is linked to gene silencing and histone deacetylases (HDACs) are considered as transcriptional co-repressors [2]. Only recently, a more general role of histone deacetylation during transcriptional regulation is emerging [3]. The use of chemical HDAC inhibitors and knock out/down technologies resulted in the identification of an increasing number of target genes controlled by histone acetylation in different cellular contexts [4]–[6]. Based on sequence homology, mammalian HDACs have been divided into four classes, of which class I enzymes seem to fulfil more basic cellular functions whereas class II enzymes are thought to play specialised cell-type specific roles. HDACs are often found to interact with proteins important for stable gene silencing *via* DNA methylation and constitution of heterochromatin, but also with transcriptional regulators mediating only transient repression. Crosstalk between histone acetylation and other epigenetic marks is an important feature of HDAC function [7]. Hence, HDACs are central components of multiple silencing complexes containing additional enzymatic activities such as DNA and histone methylation.

Recent annotation of multiple complete genomes has revealed that a large fraction of eukaryotic genomes consists of repetitive sequences, mainly derived from transposable elements (TEs) [8], [9]. Most of those sequences are remnants of once active TEs now incapable of transposition because of their host-mediated inactivation followed by subsequent functional erosion and the accumulation of mutations and deletions. However, some elements remain intact and constitute a constant threat to the integrity of the host genome. Potentially functional elements can act as insertion-mutagens *via* targeting protein coding genes or causing chromosome breakage. Even transpositionally inactive TEs have the potential to trigger illegitimate recombination and genome rearrangement, or to influence neighbouring genes by causing alternative splicing, premature termination, and modulating gene expression patterns [10], [11]. Recent estimations suggest that TEs provoke around 0.1% (in humans) or 10–12% (in mice) of all spontaneous germ line mutations, and an increasing number of reports document the contribution of somatic transposon activity to pathological situations [11]–[13]. Consequently, organisms were challenged to evolve multiple lines of defence to restrict TE activity, targeting crucial steps in their life cycle [11], [14]. One of the most efficient host-directed strategies is to block TE transcription. This is achieved applying epigenetic mechanisms such as DNA methylation, modification of chromatin structure, and the action of small RNAs [13], [15]–[19]. Different subclasses of small RNAs (siRNAs, rasiRNAs and piRNAs) seem to guide and target the silencing machinery, while chromatin modifiers and DNA methyltransferases (DNMTs) accomplish the transformation into a heterochromatic, transcriptionally silent state [20]. Numerous proteins, involved in the establishment, maintenance and read-out of DNA methylation and chromatin modification patterns, are crucial for TE silencing in germline and somatic cells [13]. However, the exact contribution of DNA methylation, chromatin remodelling, modification of histones and their interplay has not been completely resolved. Analysis in several model organisms revealed that the different silencing-mechanisms are of varying importance depending on the host organism. Some (e.g. yeast and Drosophila) lack an efficient DNA methylation machinery and therefore depend on alternative silencing pathways, while others (e.g. mammals and plants) widely use DNA methylation in concert with other mechanisms to silence TEs [18], [21].

Despite the tight collaboration of multiple silencing layers, numerous TEs maintain their capacity to escape host-mediated silencing under certain conditions. Different types of internal and external stimuli are able to trigger TE activity in a wide range of organisms. Many of those stimuli can be subsumed as cellular stress and the correlation between TE activity and stress has been in the focus of interest for several decades [22], [23]. Recent examples of mammalian TEs, showing increased transcriptional activity upon external stimuli, include members of all major TE orders: SINEs [24], [25], LINEs [26], and LTR elements [27].

To better understand the role of histone acetylation in transcriptional regulation of TEs in mammalian somatic cells, we monitored the expression of selected, potentially active TEs in mouse fibroblasts with impaired histone deacetylase capacity. In our present study we found that chemical inhibition of class I and II HDACs, but not genetic inactivation of HDAC1 alone, resulted in local hyperacetylation and expression of a defined subset of VL30 LTR retrotransposable elements. Furthermore, we show that not only histone acetylation, but histone phosphorylation in concert with acetylation (phosphoacetylation) was triggering transcriptional induction of VL30 elements. We suggest a model, in which external stress signals cause the activation of MAP kinase pathways, ultimately leading to the phosphoacetylation of histones and efficient transcription of VL30 elements.

## Results

To assess the epigenetic control of TEs with respect to histone acetylation, we treated mouse 3T3 fibroblasts with the deacetylase inhibitor trichostatin A (TSA), a compound efficiently inhibiting class I and II HDACs and the DNA methyltransferase inhibitor 5′-Aza-2′-deoxycytidine (Aza-dC). Pilot studies had shown that loss of the class I deacetylase HDAC1 in fibroblasts resulted in significantly reduced total cellular HDAC activity, suggesting a prominent role of this enzyme in histone acetylation in this cell type (data not shown). Therefore we also included immortalised HDAC1-deficient fibroblasts (HDAC1^−/−^) in this study.

### Effects of HDAC1 depletion, TSA, and Aza-dC treatment on mouse fibroblasts

**Figure 1:**
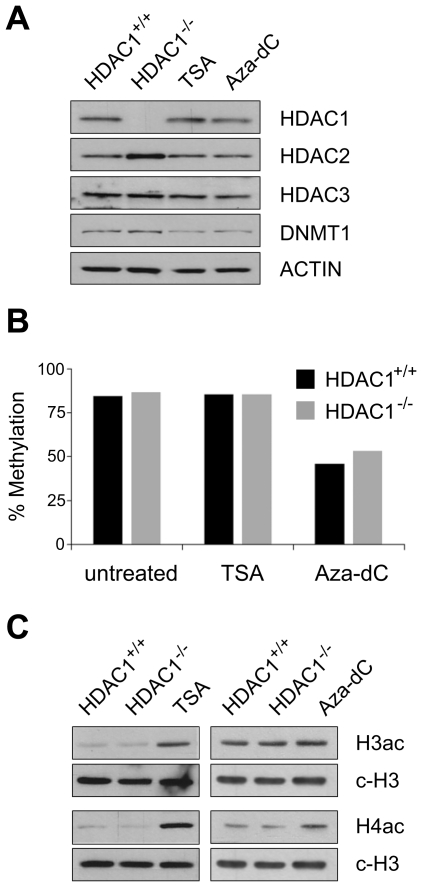
Effects of HDAC1 depletion, TSA treatment, and Aza-dC treatment on DNA–methylation and histone acetylation patterns in mouse fibroblasts. Mouse wildtype fibroblasts were left untreated (HDAC1^+/+^) or treated with TSA (166nM; 24h for (A) and (B); 3h for (C)) or Aza-dC (1µM for 24h; 24h recovery). In addition untreated HDAC1 deficient fibroblasts (HDAC1^−/−^) were included in these experiments. (A) Western blot analysis of chromatin modifying enzymes. Proteins were extracted, separated by SDS-PAGE and analysed with antibodies specific for HDAC1, HDAC2 and DNMT1. Equal loading was controlled with an antibody raised against β-ACTIN. (B) DNA methylation of IAP retrotransposons in HDAC1^+/+^ (black bars) and HDAC1^−/−^ (grey bars) fibroblasts. Both cell lines were treated with TSA or Aza-dC as described above. Average DNA methylation levels of three CpG sites within the LTR were determined employing the Ms-SNuPE technique. (C) Analysis of histone acetylation patterns. Histones were extracted and analysed on Western blots using H3ac and H4ac specific antibodies. Equal loading was monitored with an antibody recognising the C-terminus of histone H3 (c-H3).

To confirm that our experimental setup indeed perturbed epigenetic states in mouse fibroblasts, we performed the following experiments: firstly, we monitored the expression levels of proteins targeted by chemical or genetic inhibition. Protein expression levels of the class I deacetylases HDAC1, HDAC2, HDAC3 and the maintenance DNA methyltransferase DNMT1 were surveyed *via* Western blot. As shown in Figure 1A, loss of HDAC1 in immortalized fibroblasts led to upregulation of HDAC2, its closest homologue. This compensatory mechanism may buffer some effects caused by the loss of HDAC1, but still total cellular HDAC activity was diminished by about 30% in HDAC1^−/−^ compared to HDAC1^+/+^ cell lines (data not shown), demonstrating an important enzymatic function of HDAC1 in fibroblasts. In our experimental setup TSA treatment for 24h did not influence expression of HDAC1, HDAC2 or HDAC3, whereas DNMT1 levels were slightly decreased. A reduction of DNMT1 levels after histone deacetylase inhibitor treatment has been previously reported [28], [29]. Aza-dC treatment resulted in slightly reduced amounts of DNMT1, consistent with earlier reports showing that Aza-dC can trap DNMT1 by covalently linking the enzyme to DNA [30] or lead to proteasomal degradation of the DNMT1 protein [31]. Protein expression of HDAC1, HDAC2 and HDAC3 remained unaffected.

Next, we evaluated effects on the biological targets of HDACs and DNMT1, *i.e.* the acetylation states of histones and CpG methylation levels of DNA. To examine CpG methylation levels at defined targets we employed the MS-SNuPE technique [32]. We quantified DNA methylation levels of three CpG sites within the regulatory LTR region of an IAP retrotransposons, highly methylated in somatic cells [33]. Whereas neither loss of HDAC1, nor chemical inhibition of HDACs with TSA for 24h affected levels of DNA methylation, Aza-dC treatment for 24h severely reduced DNA methylation of the IAP LTR region to 50% (Figure 1B).

Furthermore, we analysed histone acetylation levels in wildtype fibroblasts, either untreated (HDAC1^+/+^) or treated with TSA and Aza-dC and HDAC1-deficient cells (HDAC1^−/−^). To that end we extracted histones and performed Western blot analyses using antibodies recognising acetylated histones. Previous reports had shown that in murine embryonic stem cells genetic inactivation of Hdac1 was accompanied by hyperacetylation of histones H3 and H4, detectable with antibodies recognising acetylated histone H3 (H3ac) and acetylated histone H4 proteins (H4ac) [34]. In our fibroblast system, however, we failed to detect global hyperacetylation of histones in HDAC1^−/−^ cells as compared to HDAC1^+/+^ cells using such H3ac or H4ac antibodies (Figure 1C); only when we probed the blots with antibodies specifically recognising the acetylation state of individual lysine residues, we detected weak hyperacetylation occurring at defined residues (data not shown). In contrast to this limited site-specific response, inhibition of all class I and II HDACs with TSA led to a robust and global hyperacetylation detectable with H3ac and H4ac antibodies. Finally, interfering with DNA methylation by Aza-dC treatment moderately enhanced global H3 and H4 acetylation levels (Figure 1C).

### Histone acetylation of transposon-associated chromatin

**Figure 2:**
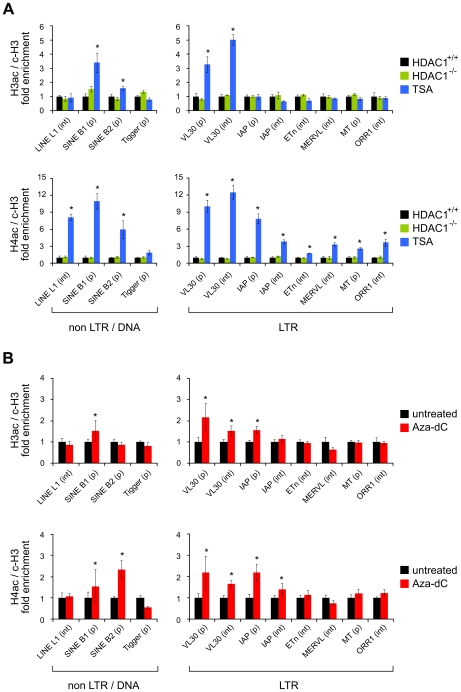
Histone acetylation states of transposable elements upon HDAC1 depletion, TSA treatment, and Aza-dC treatment. The histone acetylation state of chromatin associated with defined transposable elements was determined *via* ChIP technique using antibodies specifically recognising acetylated histone H3 (H3ac) and acetylated histone H4 (H4ac). Values were normalised to the nucleosome density as determined *via* ChIP using antibodies recognising the C-terminal region of histone H3 (c-H3) and are given relative to the acetylation levels in untreated fibroblasts. Control experiments employing unspecific IgG antibodies consistently led to negligible amounts of precipitated material (data not shown). (A) Histone H3 and H4 acetylation levels of a variety of transposable elements in HDAC1^−/−^ (green bars) and TSA (166nM; 24h; blue bars) treated fibroblasts as compared to untreated HDAC1^+/+^ (black bars) fibroblasts. (p) promoter region ±500bp of transcriptional start site; (int) internal region. N = 3; ± SEM *p<0.05 (paired, one sided student's t-test). (B) Corresponding acetylation levels of transposable elements in Azd-dC treated fibroblasts (1µM; 24h, 24h recovery; red bars). N = 5; ± SEM *p<0.05.

After defining global effects of HDAC and DNMT inhibition in our cell system, we moved on to specifically address the role of histone acetylation in the regulation of TEs. We analysed the acetylation state of chromatin associated with defined TEs in wildtype fibroblasts without treatment (HDAC1^+/+^), or treated either with TSA or Aza-dC, and HDAC1-deficient fibroblasts (HDAC1^−/−^). To monitor their acetylation status, we performed chromatin-immunoprecipitation (ChIP) experiments with H3ac and H4ac antibodies. Furthermore, we included antibodies recognising the C-terminal part of histone H3 to survey nucleosome occupancy and unspecific rabbit IgGs to control the specificity of immunoprecipitation reactions. Quantitative Real-Time PCR analyses allowed the quantification of H3 and H4 acetylation levels associated with a representative selection of ten murine TEs, including a DNA transposon (Tigger), non-LTR retrotransposons (LINEs, SINEs), and LTR retrotransposons (VL30, IAP, ETn, MERVL, MT, ORR1) (Table 1). We chose LINEs and SINEs, because they constitute the majority of murine TEs and populate the genome in more than 660,000 and 1.5 million copies, respectively. LTR elements are less abundant, but harbour the most active TEs within the mouse lineage; MERVL, MT and ORR1 elements are expressed during early embryogenesis [35] and VL30, IAP and ETn elements have been responsible for a number of germ line transposition events [12]. The transposon Tigger was included as representative of the class of DNA elements, which is functionally extinct in vertebrates. As shown in Figure 2A (upper panel), loss of HDAC1 did not influence histone H3 acetylation at any transposon type analysed as compared to wildtype cells. In contrast, TSA treatment was sufficient to specifically enhance H3 acetylation at SINE B1 and B2 elements and VL30 LTR elements, the latter ones near the promoter as well as throughout the body of the element. Histone H4 acetylation of TE-associated chromatin was analysed similarly in a subsequent series of experiments. Again, loss of HDAC1 did not alter histone acetylation at tested transposons, but TSA-induced inhibition of all class I and II HDACs led to increased H4 acetylation at all repeat families investigated (Figure 2A; lower panel). Thus, TSA treatment exhibited differential effects on histone H3 and histone H4 acetylation of TEs. Whereas H3 histones were found hyperacetylated at specific elements only, H4 hyperacetylation occurred as a global response. Interestingly, the elements hyperacetylated at H3 upon TSA treatment, SINEs and VL30, also showed highest enrichment of H4 acetylation compared to other TEs.

**Table 1:** Transposable elements analysed in this study.

Class	Order	Superfamily	Family	Copy Number	PCR Amplicon ChIP
I	LINE	L1	-	599000*	internal
I	SINE	7SL	B1	564000*	promoter
I	SINE	tRNA	B2	348000*	promoter
II	TIR	Tc1-Mariner	Tigger	24000*	promoter
I	LTR	ERV1	VL30	150–200°	promoter / internal
I	LTR	ERV2	IAP	1000°	promoter / internal
I	LTR	ERV2	ETn	300–400°	internal
I	LTR	ERV3	MERVL	200°	internal
I	LTR	ERV3	MT	13000–50000#	promoter
I	LTR	ERV3	ORR1	10000–38000#	internal

Classification of TEs, estimated copy numbers and approximate location of the amplicons used to survey chromatin modification states *via* chromatin immunoprecipitation (ChIP). Nine out of the ten elements are retrotransposons and propagate *via* a RNA intermediate (Class I), one is a DNA transposon (Class II) [88]. The copy numbers are estimates from different references (*[9]; °[12]; #[89]). The location of the amplicon was defined as promoter specific, if both primers were located within ±500bp of the transcriptional start site, otherwise as internal.

Next, we analysed histone acetylation levels upon loss of DNA methylation. Incubation of cells with Aza-dC increased H3 acetylation at SINE B1 and VL30 elements (Figure 2B, upper panel), although to a lesser extent than TSA. Furthermore, IAP element promoter sequences exhibited slightly increased H3 acetylation levels in response to Aza-dC. Finally, Aza-dC treatment resulted in elevated H4 acetylation levels of SINEs, VL30 and IAP elements (Figure 2B; lower panel).

### Transcriptional activation of transposable elements

**Figure 3:**
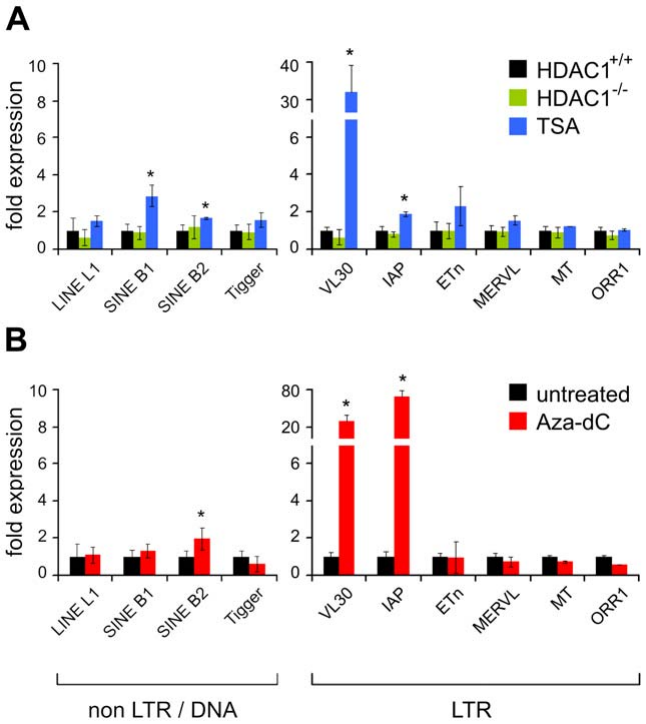
Effect of TSA and Aza-dC treatment on the expression of transposable elements. (A) Logarithmically growing fibroblasts show increased mRNA expression levels of VL30 LTR elements when treated with TSA. Expression levels were determined with qRT-PCR. Values are normalised to HPRT expression and are shown relative to the expression in untreated HDAC1^+/+^ fibroblasts. Treatment as in Figure 2. N = 3; ± SD *p<0.05. (B) Aza-dC causes high level induction of VL30 and IAP LTR element expression. N = 3; ± SD *p<0.05.

Since hyperacetylation of histones in general correlates with transcriptional activation, we tested whether increased histone acetylation levels were mirrored by changes in transcriptional activity of TEs. Therefore we monitored mRNA levels of the ten mouse transposons, listed in Table 1, in untreated wildtype fibroblasts (HDAC1^+/+^), TSA or Aza-dC treated wildtype cells, and HDAC1-deficient fibroblasts (HDAC1^−/−^) by real-time PCR analyses. We also analysed the expression of another LTR element, MuLV, which comprises endogenous as well as exogenous family members [36] (Figure S1). As shown in Figure 3, upper panel and Figure S1, loss of HDAC1 did not influence TE expression. In contrast, general HDAC inhibition *via* TSA strongly increased VL30 element expression in this fibroblast cell line (Figure 3, upper panel), as well as in other commonly used fibroblast cell lines such as Swiss 3T3 and NIH/3T3 (data not shown). Enhanced activity of VL30 elements correlated with hyperacetylation of VL30 chromatin upon TSA treatment. This suggests that modulation of histone acetylation regulates VL30 element activity. Expression of SINE B1, the second element showing H3 and H4 hyperacetylation after TSA treatment, was modestly increased. Besides IAPs showing a moderate two-fold increase, all other TE candidates investigated did not exhibit major changes in transcriptional activity. This expression pattern mirrors the unchanged local acetylation state of histone H3, but not the one of histone H4 acetylation, which was elevated upon TSA treatment. Therefore, H3 acetylation of the transposons analysed correlates with transcriptional activity, whereas H4 hyperacetylation is not sufficient to trigger transcriptional activation.

The role of DNA methylation in transposon silencing is well established and it has been shown earlier that loss of DNA methylation in somatic cells correlates with VL30, IAP and MuLV expression [37]–[39]. Indeed, Aza-dC treatment in fibroblasts caused highly elevated VL30, IAP and MuLV mRNA levels, but had no effect on other TEs tested (Figure 3, lower panel and Figure S1). However, it was surprising that the majority of murine elements tested could not be reactivated with Aza-dC. This finding suggests that those transposons are not permissive for transcriptional activation in our fibroblast system, either because they lack regulatory features necessary for efficient transcriptional activation or additional mechanisms contribute to their silencing [40]. ChIP analyses of TE-associated chromatin showed that two elements that were highly expressed upon Aza-dC treatment (VL30 and IAP elements) also gained hyperacetylation after DNMT inhibition (Figure 3, lower panel). SINE B1 and SINE B2 elements, both showing increased acetylation upon Aza-dC treatment, were not or only modestly activated.

In summary, we could establish a correlation between the transcriptional activity of TEs and the acetylation state of associated chromatin. In particular histone H3 acetylation correlated well with increased expression of certain TEs. Furthermore, we identified VL30 elements as the single candidate transposon efficiently regulated *via* acetylation of associated chromatin.

### Transposition frequency

Retrotransposition is a complex process depending on multiple steps, each of which can be targeted by regulatory mechanisms [11]. Alterations in RNA stability, translation efficiency, nuclear shuttling, editing of template RNA, or integration efficiency can have severe impact on the rate of successful transposition.

Treatment of cells with the HDAC inhibitor TSA has strong effects on the chromatin state. Since chromatin constitutes the substrate for the last step of transposition, *i.e.* integration, we tested whether histone modification patterns might influence transposition efficiency, independent of TE transcription. Genetic screens in yeast have uncovered numerous host factors either restricting or enhancing transposon activity [41]–[45]. Strikingly, a substantial fraction of the proteins identified has a role in chromatin biology; for instance, SIN3 and RPD3, both major components of yeast HDAC complexes, were found to restrict Ty1 activity and modulate target site selection. Interestingly, SIN3 and RPD3 do not act on the transcriptional level, as they do not alter Ty1 mRNA levels [45].

To test whether changed histone acetylation levels induced by TSA treatment might have similar effects in mammalian cells, we determined transposition levels of four mammalian retroelements in untreated and TSA treated cells. To this end we took advantage of an experimental system described earlier [46]–[48], in which episomally delivered retrotransposons are marked with a neo-transgene, which gets activated only after its successful retrotransposition (Figure S2A). Following this approach we quantified the transposition efficiency of murine IAP and MusD LTR retrotransposons, and murine and human L1 elements in untreated and TSA treated cells. In order to avoid potential interference with endogenously transcribed IAP, MusD and L1 elements, we performed the experiments in the human U2OS osteosarcoma cell line. In addition, we monitored transcript levels of episomally delivered retroelements *via* real-time PCR (Figure S2B). As presented in Figure S2C, TSA treatment led to a 2- to 4-fold increase in transposition rates, a moderate increase compared to findings reported elsewhere, where mutation of TE restricting factors in yeast led to an increase between 5 and several hundred times [45]. In our system, enhanced transposition was accompanied by a 2- to 3-fold increase in TE mRNA abundance. These data suggest that TSA treatment led to enhanced expression of marked elements, which in turn resulted in increased transposition rates. Therefore, under our experimental conditions, inhibition of deacetylases by TSA was not sufficient to influence TE transposition rates independent of TE expression, suggesting transcription as the rate-limiting factor for transposition.

### Chromosomal origin and structural features of reactivated transposable elements

In contrast to single copy genes, TEs exist in multiple copies dispersed throughout the genome. Individual elements within a TE family are different in respect of genomic environment, influencing chromatin status and transcriptional potential. Furthermore, transposons are under high selective pressure and mutations mediated by host and/or transposon-intrinsic factors lead to the generation of pools of elements with individual sequence specificities within given TE families. Therefore, a family of elements is a collection of similar, but heterogeneous individuals, differing in external and internal features.

**Figure 4:**
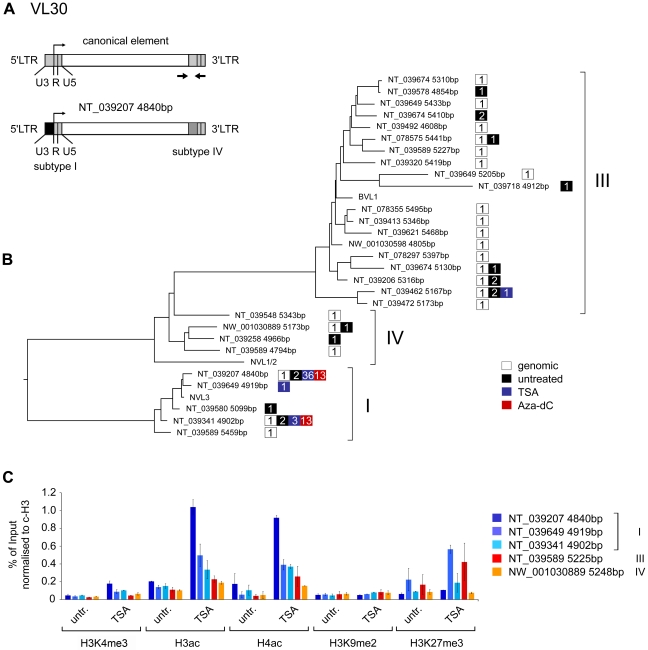
Characterisation of transcribed VL30 elements reveals preferential expression of defined VL30 elements upon stimulation. (A) Schematic view of a canonical VL30 retrotransposon. Two similar LTRs flank an internal region without functional open reading frame (upper part). Some elements have a hybrid structure and are flanked by LTRs belonging to different subtypes (lower part). Primers used to amplify fragments of genomic and transcribed VL30 elements from untreated, TSA and Aza-dC treated fibroblasts are indicated as black arrows. (B) To characterise the genomic VL30 elements identified as *bona fide* transcriptional sources, their 5′LTR sequences were aligned (using the Megalign program with the following settings: Clustal W, gap penalty 15, gap length penalty 6.66, delay divergent seqs 30%, DNA transition weight 0.5, DNA weight matrix IUB) and a phylogenetic tree was generated. The LTR sequences fall into three clades. The affiliation to one of the clades is determined by the structure of the highly polymorphic U3 region. While many VL30 elements identified in untreated fibroblasts are associated with a subtype III U3 region, TSA and Aza-dC treatment leads to preferential expression of elements with a type I U3 region. Coloured squares indicate the source, where the VL30 element has been isolated from (white, genomic DNA; black, untreated fibroblasts; blue, TSA treated fibroblasts (166nM; 24h); red, Aza-dC treated fibroblasts (1µM; 24h; recover 24h), numbers indicate the transcriptional activity of the respective elements (*i.e.* number of clones mapping best to this genomic locus). NVL3, BVL1, NVL1/2 serve as reference elements with known U3 regions and are described elsewhere [85]–[87]. (C) To survey the chromatin signature of individual VL30 elements, ChIP experiments in logarithmically growing fibroblasts untreated or treated for 12h with TSA (166nM) were performed. H3K4me3, H3ac and H4ac antibodies were used to survey the enrichment of active histone marks, H3K9me2 and K3K27me3 antibodies to measure levels of repressive marks. Values were normalised to local histone occupancy as determined with ChIP experiments using the c-H3 antibody and are given as percentage of Input. Primers targeting the upstream regions and 5′LTRs of individual elements allowed determining histone modification levels of five specific VL30 elements. NT_039207 4840bp is an element highly expressed upon TSA treatment. NT_039649 4919bp and NT_039341 4902bp are two additional elements found expressed in TSA treated fibroblasts. Those three elements are associated with a U3 subtype I LTR region. NT_039589 5224bp and NW_001030889 5248bp are equipped with subtype III and IV U3 regions, respectively. N = 2; ± SD.

Upon external signals increased transcriptional activity could originate from the upregulation of all TEs within a family, of a subgroup of elements exhibiting specific features, or even a single copy with unique intrinsic properties. In order to distinguish between these different scenarios, we aimed to determine the chromosomal origin of VL30 and IAP derived transcripts detected in untreated, TSA and Aza-dC treated wildtype fibroblasts. RNA from three independent biological samples each (untreated, TSA, Aza-dC) was collected, reversely transcribed, and subjected to PCR amplification. To amplify IAP derived transcripts, primers annealing in the highly conserved *gag* region were used (Figure S3A). For VL30 transcript amplification primers within the 3′ UTR and 3′LTR R region were chosen (Figure 4A). To exclude a bias due to individual PCR reactions, six independent PCR reactions for each condition were pooled, PCR products were cloned and sequenced. As control, the same procedure was also performed with genomic DNA as template. This approach allowed us to amplify chromosomal copies of both candidate elements and to estimate the diversity of IAP and VL30 elements in our cell system.

Each sequenced clone corresponding to an individual transcribed (or genomic) TE was then analysed as follows: to identify genomic elements serving as potential sources of transcription, sequences were blasted against the annotated murine genome (http://www.ncbi.nlm.nih.gov/blast/Blast.cgi). Genomic sequences exhibiting at least 98% identity to the cloned sequences were considered to show sufficient homology to serve as potential sources of transcription and were analysed further. If more than one genomic sequence showed identity >98%, the sequence with the highest E-value was chosen. That way ∼90% of the cloned sequences were assigned to distinct genomic elements (IAP: 115 of 127 clones; VL30: 111 of 122 clones). Individual characteristics, such as chromosomal localisation and length were examined for each element as summarised in Table S1. Next, we defined whether the transcribed IAP and VL30 elements were derived from multiple genomic sources, a small number of elements, or even unique elements. Therefore, we analysed the diversity of the identified genomic TEs, constituting *bona fide* sources of transposon transcription. Following this approach, each IAP transcript could be assigned to a different genomic element, indicating that IAP transcripts originated from multiple loci throughout the genome (see chromosomal origins and individual features of elements listed in Table S1).

In untreated cells, VL30 transcripts were of heterogeneous origin, with a broad level of sequence diversity similar to the chromosomal VL30 elements that were amplified from genomic DNA. In contrast, upon TSA and Aza-dC treatment, VL30 transcripts could be assigned to only four and two genomic loci, pointing towards a limited number of VL30 elements serving as source of expression. TSA treatment led to the accumulation of transcripts related to the genomic loci NT_039207 4840bp on chromosome 2 (36 of 41 sequences), NT_039341 4902bp on chromosome 6 (3 of 41), NT_039462 5167bp on chromosome 8 (1 of 41), and NT_039649 4919bp on chromosome 17 (1 of 41). Aza-dC treatment resulted in transcripts similar to NT_039207 4840bp on chromosome 2 (13 of 26 sequences) and NT_039341 4902bp on chromosome 6 (13 of 26) (Table S1), Alignment of VL30 sequences directly obtained after cloning and sequencing further showed that transcripts assigned to the genomic locus on chromosome 2 (NT_039207 4840bp) were almost identical, indicating a single locus as transcriptional source. In contrast, transcripts assigned to the locus on chromosome 6 (NT_39341 4902bp) were slightly divergent, demonstrating that they originate from a group of related VL30 elements, of which only one copy is annotated in the published mouse genome (data not shown).

The long terminal repeat regions of LTR elements are enriched with regulatory sequences important for transcriptional regulation. Therefore, we determined the structure of the LTR regions associated with expressed IAP and VL30 elements in more detail. First, we analysed the LTRs associated with transcribed IAP elements, which revealed a marked length polymorphism between different LTRs: the shortest LTR, identified with an IAP element expressed in untreated fibroblasts, comprised 322bp, whereas the longest LTR, found in an IAP element expressed after Aza-dC treatment, was 456bp long. Alignment of IAP LTR sequences disclosed that this difference was due to the presence or absence of nucleotides in a highly variable CT-dinucleotide-rich stretch within the R region of the LTR (Table S1 and Figure S3B). The region encoded a varying number of tandem repeat sequences containing a 13bp core sequence (TCTCTCTTGCTTC), previously described as polymorphic marker for different IAP subtypes [49]. Interestingly, the average length of this stretch was significantly longer in LTRs associated with IAPs expressed upon Aza-dC treatment (Figure S3C), indicating that upon loss of DNA methylation an increased number of tandem repeats might be favourable for expression.

As shown in earlier studies the structure of the VL30 LTR region is crucial for stimulus mediated activation of VL30 expression (reviewed in [27]). Based on their highly divergent U3 regions, VL30 LTRs have been classified into four distinct subtypes: I, II, III and IV [50]. To allocate the VL30 elements isolated in our transcript analysis to the four subtypes, we isolated the 5′LTR sequences from the genomic sequences, aligned them together with VL30 LTR reference sequences (NVL3: subclass I, BVL1: subclass III, NVL1/2: subclass IV) and generated a phylogenetic tree. As depicted in Figure 4B, all elements including the references were located in three distinct clades corresponding to subclass I, III and IV elements. Elements expressed in untreated cells mainly comprehend subtype III containing LTRs, which also associate with most genomic elements (Figure 4B and Table S1). As described above, transcripts arising upon TSA and Aza-dC treatment, mainly originate from two types of loci: one VL30 insertion on chromosome 2 (NT_039207 4840bp) and several other loci showing high similarity to another element on chromosome 6 (NT_039341 4902bp). Interestingly, whereas the VL30 element on chromosome 6 is flanked by LTRs containing subtype I U3 regions at both ends, the element located on chromosome 2 exhibits an unusual structural feature: it is associated with two different LTRs, a 5′ LTR containing a U3 region belonging to subtype I, but a 3′ LTR containing a U3 region of subtype IV (Figure 4A, lower part). Transposon transcription depends on the 5′ LTR, therefore we conclude that expression of this VL30 element is controlled by LTR sequences comprising a subtype I U3 region. 40 out of 41 transcripts isolated from TSA treated fibroblasts were associated with a LTR containing a subtype I U3 region, suggesting that this LTR design might be favourable for VL30 expression upon TSA treatment. However, 36 of those 40 were derived from a single genomic source. Therefore we wanted to clarify if increased VL30 transcription upon TSA treatment is exclusively caused by activation of this particular locus mirroring a locus specific phenomenon or if it reflects a general feature of subtype I elements. We surveyed the chromatin signature at the 5′LTRs of five genomic VL30 elements *via* ChIP using flanking primers: the highly responsive hybrid element on chromosome 2 (NT_039207 4840bp), two other subtype I elements (NT_039649 4919bp, NT_039341 4902bp), one subtype III (NT039589 5225bp) and one subtype IV element (MW_001030889 5248bp) (Figure 4C). Interestingly, we detected increased levels of H3K4me3 (a mark for active promoters) upon TSA treatment at all five VL30 elements, with the highest levels at the hybrid element, followed by the two other subclass I elements (Figure 4C). The subclass III and IV element showed the lowest response. A similar pattern could be observed for H3ac and H4ac. In addition we monitored the repressive histone mark H3K9me2, which was low in all elements and remained unchanged upon TSA treatment. Another repressive mark, H3K27me3 showed variable levels with no clear correlation with VL30 subtypes. Together these data suggest an increased responsiveness to activation of the hybrid element, followed by other subclass I elements and finally subclass III and IV elements.

### Regulation of VL30 elements by a dual histone modification mark

Based on the finding that VL30 elements respond to histone acetylation, we examined the potential activation mechanism for VL30 elements in detail. In the recent past VL30 elements have been studied extensively and others showed that VL30 elements expression is highly sensitive to a plethora of internal and external stimuli [27], [39], [51]. Interestingly, many of those stimuli can be described as internal or external stress in the broadest sense, and a considerable number among them is able to trigger signalling cascades such as the ERK or p38 pathways. Signalling *via* these pathways ultimately leads to phosphorylation and activation of several transcription factors but also to phosphorylation of histone H3.

**Figure 5:**
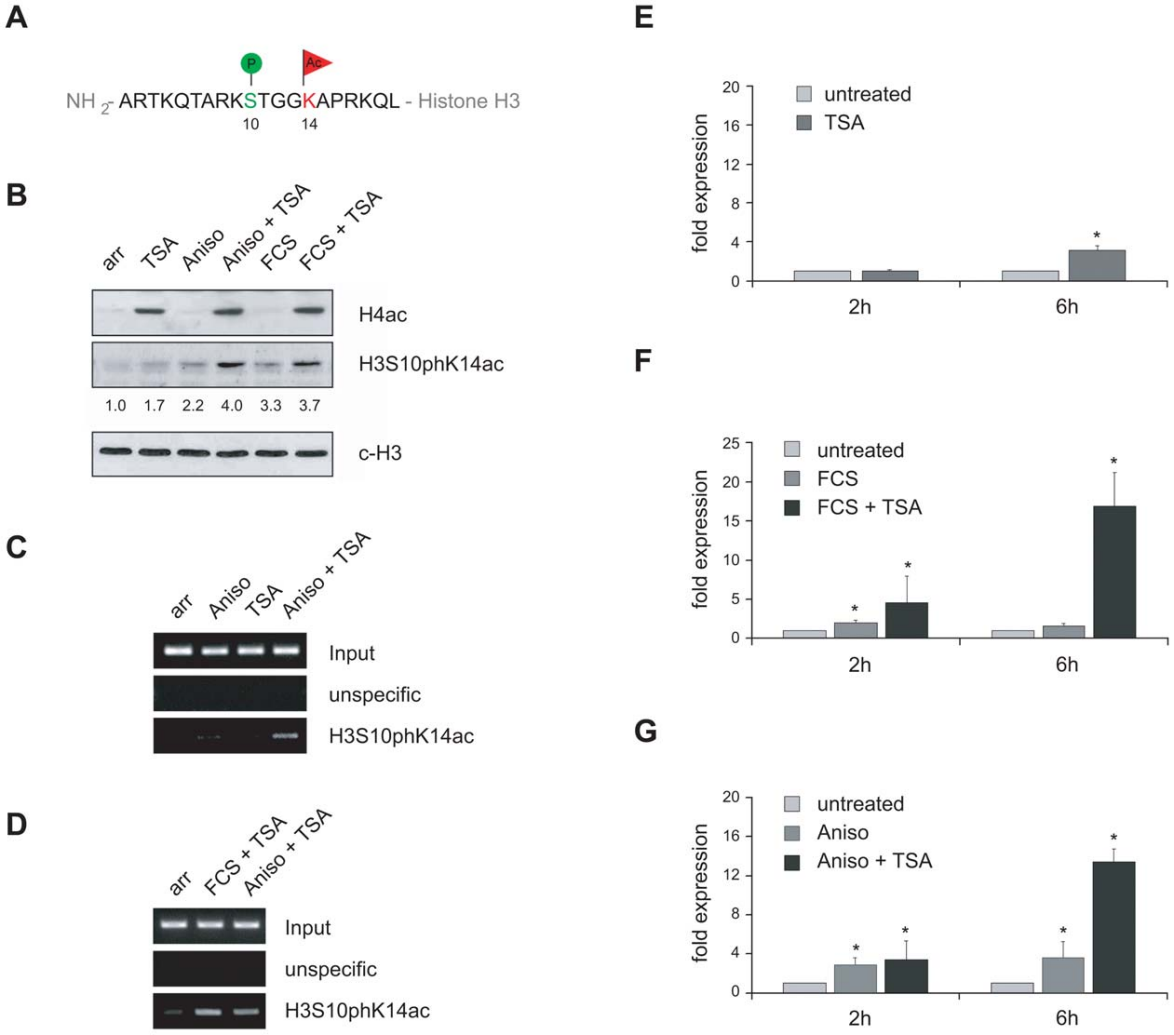
VL30 expression correlates with the formation of a phosphoacetylation mark at chromatin associated with VL30 LTR sequences. (A) Schematic view of a histone H3 tail carrying a so-called phosphoacetylation mark, *i.e.* concomitantly phosphorylated serine 10 (H3S10ph) and acetylated lysine 14 (H3K14ac). (B) Phosphoacetylation of histone H3 in response activation of the MAP kinase pathway and TSA treatment in quiescent Swiss 3T3 fibroblasts. Serum deprived Swiss 3T3 fibroblasts were left untreated (arr) or treated for 2 hours with 166nM TSA, 189nM anisomycin (Aniso) or 20% v/v fetal calf serum (FCS) alone or combinations of anisomycin and TSA or FCS and TSA. Western blot analysis was performed with antibodies directed against acetylated histone H4, H3S10pK14ac and an antibody against the C-terminus of histone H3 (c-H3) as loading control. H3S10pK14ac The H3S10pK14ac signals were determined by densitometric scanning, normalised to c-H3 signals and are shown relative to the value of untreated cells taken arbitrarily as 1. (C,D) The LTR region of VL30 elements is associated with histones carrying a phosphoacetylation mark only upon MAP kinase pathway activation together with HDAC inhibition. ChIP experiments using an H3S10pK14ac specific antibody reveals the presence of phosphoacetylated histones at VL30 LTR regions after anisomycin (189nM; 3h) or FCS (20%; 3h) and TSA (166nM; 3h) treatment (Aniso + TSA; FCS + TSA). (E–G) Combinatorial treatment efficiently activates VL30 element expression in quiescent Swiss 3T3 fibroblasts. Single treatment with TSA (166nM), FCS (20%) or anisomycin (189nM) (dark grey bars) leads to a modest induction compared to VL30 mRNA levels in untreated cells (light grey bars). In contrast, combinatorial treatment with anisomycin or FCS and TSA robustly increases VL30 expression after 6h (black bars). VL30 expression levels were determined with qRT-PCR; values are normalised to HPRT levels and given relative to VL30 levels in untreated cells. N = 3; ± SD *p<0.05.

Phosphorylation of serine 10 at histone H3 (H3S10p) during interphase is a rare event that correlates with the activation of specific target genes [52], [53]. Importantly, the presence of the H3S10ph mark at promoter-associated chromatin coincides with acetylation of neighbouring lysines K9 and K14 leading to a dual histone modification termed phosphoacetylation [54]–[56] (Figure 5A). Histone H3 phosphoacetylation correlates with the activation of the so called “immediate early” genes c-fos and jun-B, as well as late inducible genes such as HDAC1 [57].

The finding that the HDAC inhibitor TSA was sufficient to highly induce VL30 expression in logarithmically growing fibroblasts, where ERK pathways are activated through the action of growth factors, prompted us to ask whether both stimuli, histone acetylation and histone phosphorylation, were required for full VL30 activation. To answer this question, we performed all following experiments with serum-arrested Swiss 3T3 fibroblasts. Serum withdrawal arrests cells in G_0_ and eliminates mitotic H3 phosphorylation, a modification that targets the majority of histone H3 molecules during mitosis and is associated with chromatin condensation. Furthermore, the absence of the serum-dependent activity of different kinase pathways allows to selectively activate specific signalling cascades, thereby triggering H3 phosphorylation. To induce the ERK pathway in fibroblasts, cells were arrested in G_0_ for 72h and then treated with foetal calf serum (FCS). Alternatively, anisomycin (Aniso) was used to activate the p38 stress pathway. Additionally cells were treated with TSA to induce hyperacetylation.

To analyse simultaneous phosphorylation and acetylation of histone H3 we used an antibody that recognises histone H3 only in the presence of the dual H3S10phK14ac mark (Figure 5A, Figure S4). Figure 5B shows the effect of different compounds on histone H4 acetylation and histone H3 phosphoacetylation in G_0_ arrested Swiss 3T3 fibroblasts. As expected, TSA led to the accumulation of hyperacetylated histones as detected by the H4ac antibody; H3 phosphoacetylation was low in untreated and TSA treated G_0_ cells and slightly induced upon Aniso or FCS treatment. Combined treatment with Aniso/TSA or FCS/TSA led to enhanced phosphoacetylation of histone H3.

It was shown previously that, even upon Aniso/TSA stimulation, the fraction of phosphoacetylated histone H3 is only minute [52], [58] and that the mark is localised to a restricted number of genomic loci in Aniso/TSA treated fibroblasts (Simboeck E., unpublished results). Hence we tested, if phosphoacetylated histones were associated with VL30 elements. To this end we isolated chromatin from G_0_ arrested fibroblasts that were untreated or treated with TSA, Aniso, or Aniso/TSA. We performed ChIP experiments using the H3S10ph/H3K14ac antibody or an unspecific control antibody and surveyed phosphoacetylation levels of the VL30 5′LTR regulatory region *via* semi-quantitative PCR. Strikingly, we could detect phosphoacetylated histones at VL30 elements at low levels when cells were treated with Aniso alone, and at higher levels upon Aniso/TSA treatment, but not in untreated or TSA-treated cells (Figure 5C). Additional ChIP experiments with resting cells activated with growth factors, containing serum in combination with TSA (FCS/TSA), demonstrated the presence of phosphoacetylation also upon activation of the ERK pathway, together with induction of hyperacetylation (Figure 5D). In summary, activation of the p38 or ERK pathway together with induction of hyperacetylation resulted in the establishment of a phosphoacetylation mark at VL30 LTRs. In agreement with these findings we detected an increased presence of the H3S10phK14ac mark at individual VL30 insertions in TSA-treated proliferating HDAC1^+/+^ fibroblasts suggesting a direct link between this dual histone modification and transcriptional induction of particular VL30 elements (Figure S5).

**Figure 6:**
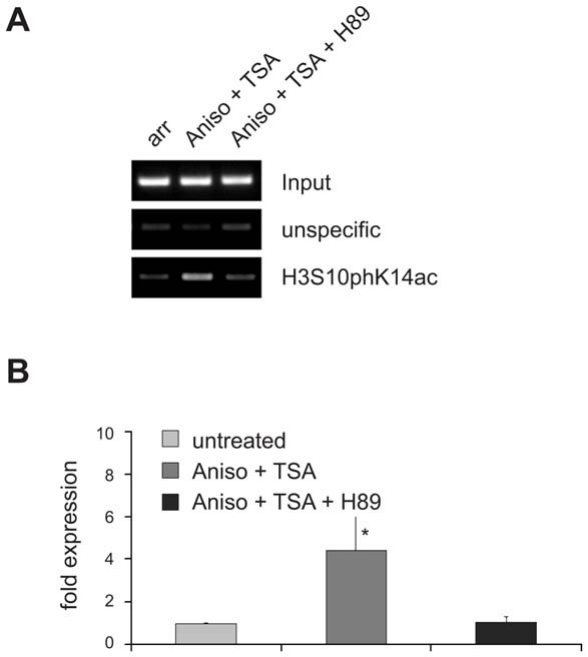
Inhibition of H3S10 phosphorylation with the protein kinase inhibitor H89 precludes transcriptional activation of VL30 elements. (A) Treatment of arrested Swiss 3T3 fibroblasts with the protein kinase inhibitor H89 (10µM), inhibiting H3S10 phosphorylation, prohibits the creation of a phosphoacetylation mark upon 1h of Aniso+TSA treatment. ChiP experiments were performed as in Figure 5C. (B) Precluding phosphoacetylation of VL30 elements with H89 also abolishes transcriptional induction upon Aniso + TSA treatment. Treatments as in (A); quantification of VL30 transcript levels were determined as in Figure 5E. N = 3; ± SD *p<0.05.

Next, we examined the effect of the different stimuli on VL30 expression. We isolated RNA from arrested 3T3 Swiss fibroblasts exposed to the aforementioned inducers, reversely transcribed them and quantified VL30 levels with Real Time PCR. Whereas single treatment of cells with TSA, Aniso, or FCS led to a modest increase in expression, combined treatment with Aniso/TSA or FCS/TSA resulted in robust induction, suggesting a synergistic effect between histone phosphorylation *via* p38 or ERK activation and histone acetylation (Figure 5E–5G). To directly test whether activation of the ERK and p38 pathways is linked to H3 phosphoacetylation and VL30 activation, we took advantage of the protein kinase inhibitor H89. This compound was used at a concentration (10µM), shown to efficiently block histone H3 phosphorylation without affecting transcription factor phosphorylation [53]. As presented in Figure 6A, inhibition of kinase activity *via* H89 abolished the formation of a phosphoacetylation mark upon Aniso/TSA treatment in arrested Swiss 3T3 fibroblasts. Equally important, H89 treatment also prevented transcriptional induction upon Aniso/TSA treatment (Figure 6B), indicating that H3S10p/H3K14ac does not only correlate with transcriptional induction mediated by Aniso/TSA treatment, but is necessary for proper VL30 expression.

**Figure 7:**
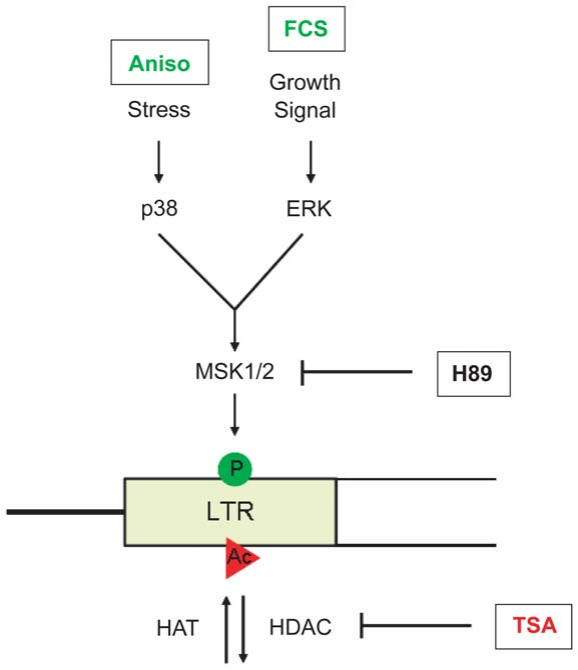
Model of VL30 element activation. Full transcriptional activation of VL30 elements is triggered by a dual histone modification mark, *i.e.* phosphoacetylation of histone H3 at serine 10 and lysine 14. Phosphorylation of serine 10 (green circle) is conveyed by activation of the p38 stress (e.g. *via* anisomycin (Aniso)) or the ERK growth factor inducible MAPK pathway (e.g. *via* serum (FCS)) ultimately leading to the activation of the MSK1/2 kinase and H3S10 phosphorylation. Histone hyperacetylation (red circle) can be induced by HDAC inhibition (e.g. *via* TSA). Combined treatment results in the formation of phosphoacetylated histones at VL30 LTRs and full transcriptional activation.

Finally, we want to propose the following model for VL30 regulation, which integrates many of the previous findings about VL30 induction: external stimuli activate the p38 (stress) or the ERK (growth signal) pathway leading to activation of effector kinases such as MSK1/2. By local phosphorylation of H3S10 at responsive VL30 elements together with the induction of hyperacetylation, a phosphoacetylation mark is established leading to full transcriptional activation (Figure 7).

## Discussion

It is well established that histone acetylation plays a fundamental role in the regulation of gene expression. This has been demonstrated on a genome wide level as well as for numerous individual genes [59]–[61]. The vast majority of work in mammals has been dedicated to gene regulation in different cancer cells [62], throughout development [6], [63] or to the silencing of retroviruses such as HIV [64], [65]. Also the role of histone acetylation in the reactivation of a silenced artificial transposon has been investigated [66], but to our knowledge no extensive study has been performed so far to specifically analyse the interrelation between histone acetylation and regulation of endogenous retroelements. This is surprising, since chemicals targeting histone deacetylases are considered as promising agents for the treatment of several human diseases and possible effects on the expression of endogenous retroelements remain largely unknown. Therefore we aimed to define the role of histone acetylation on TE activity in the mouse model system. We used an experimental system, in which cellular histone acetylation levels were shifted towards a hyperacetylated state. This was achieved using the broad HDAC inhibitor TSA. Depletion of the major histone deacetylase HDAC1 was not sufficient to induce global hyperacetylation. Interestingly, incubation of cells with the DNMT inhibitor Aza-dC also resulted in increased histone acetylation levels, once more underscoring the close link between DNA methylation and histone acetylation [7]. However, this relation seems to be asymmetric, since the maintenance of DNA methylation did not require normal histone acetylation levels.

### TE expression and histone acetylation

Having monitored global changes mediated by TSA and Aza-dC treatment, we focused our interest on the interrelation between histone acetylation and TE regulation. We determined histone acetylation levels of a representative selection of TEs in HDAC1^−/−^ background, upon TSA and Aza-dC treatment. Subsequently, we correlated these data with the expression levels of the corresponding TEs. This analysis revealed the following key findings: (1) only three out of eleven TEs tested were significantly activated by Aza-dC treatment. Inhibition of DNA methyltransferases in fibroblasts strongly enhanced the expression of IAP, VL30 and MuLV LTR elements. These data indicate that a block in DNA methylation was sufficient to activate transcription of some, but not all TEs in fibroblasts. Efficient transcriptional activation of other TEs might depend on activating cofactors absent in fibroblasts. This is consistent with the finding that many TEs exhibit tissue- or cell type-specific expression patterns [67]–[69]. Alternatively, additional layers of silencing mechanisms directed against specific TE families could be present in fibroblasts in the absence of DNA methylation.

(2) Enhanced expression roughly correlated with increased acetylation. This was obvious for VL30 elements, which were highly expressed upon TSA and Aza-dC treatment, and for IAP elements, which were induced after treatment with Aza-dC: both LTR elements exhibited hyperacetylation when activated. Remarkably, loss of DNA methylation at IAP elements was accompanied by increased acetylation, whereas HDAC inhibition by TSA did not result in IAP-associated histone hyperacetylation. This suggests a model, in which histone acetyltransferase (HAT) activity is tethered to IAPs only upon loss of DNA methylation, which otherwise might prohibit the binding of transcription factors recruiting HATs. Hence, HDAC inhibition in cells with intact DNA methylation was not sufficient to induce local hyperacetylation. The situation of VL30 element regulation was strikingly different. Inhibition of HDAC activity led to robust hyperacetylation, pointing towards a direct involvement of HDACs and HATs in the transcriptional control of VL30 elements. Finally, we observed that SINE elements gained hyperacetylation upon Aza-dC and TSA treatment, which was accompanied with mild effects on transcription. However, SINEs posses some peculiar features, making them distinct from other TEs: they are short, highly repetitive sequences, which tend to accumulate nearby and within genes and their regulatory regions. Therefore, it is difficult to unambiguously assign the increase in histone acetylation and RNA abundance to direct activation of SINE elements and not to the activation of Aza-dC- or TSA-responsive target genes containing SINE element sequences.

(3) TSA treatment led to hyperacetylation of histone H4 independent of transcriptional activity. Histone H4 specific HATs seemed to readily acetylate lysine residues of H4 when the dominant HDACs were inhibited, suggesting their regular presence at silent TEs. In contrast, acetylation of histone H3 seemed coupled with transcription, pointing towards a model, in which histone H3 acetylation might stimulate the recruitment of the transcription machinery. Alternatively, histone H3 specific HATs were recruited together with the transcription machinery. Interestingly, Koch and colleagues recently showed that although H4ac and H3ac were both enriched around the transcriptional start site, only H3ac could be used as a highly predictive marker for gene activity [60]. One might speculate that differential HAT recruitment reflects a functional difference between histone H3 and histone H4 acetylation. In addition to their role in transcription where both modifications act synergistically, H4 (de)acetylation could have other biological functions not described so far.

### Preferential expression of defined TE subfamilies

Examining IAP and VL30 transcripts in untreated fibroblasts clearly showed that expressed TEs originate from multiple loci dispersed throughout the genome. However, upon treatment with different stimuli, certain transposon subfamilies were preferentially expressed. We could show that these inducible elements harboured characteristic DNA sequence features within the LTR, the regulatory region of the element. IAPs were more efficiently activated by Aza-dC, when they carried increasing numbers of DNA sequence repeats, containing putative binding sites for the transcription factor CCAAT/enhancer-binding protein alpha (C/EBPα) (identified with the Alibaba2.1 transcription factor binding site program using the TRANSFAC 4.0 database). Strikingly, Ishihara and colleagues, who characterised IAP elements active upon irradiation of mouse acute myeloid leukaemia (AML) cells, isolated IAP elements with especially long stretches of C/EBPα binding sites [70] (Table S1, AB099818). In contrast, IAP elements responsible for recent integration events in the mouse germline [12] harboured only one TF binding site (Table S1, X04120 and AxinFu). Therefore, the availability of the transcription factor might influence the choice of which IAP subtypes are expressed in different cellular contexts.

Treatment with TSA or Aza-dC led to preferential expression of one particular VL30 element associated with a U3 subtype I LTR. This individual element also showed particularly high levels of active histone marks (H3K4me3, H3ac, H4ac, H3S10phK14ac) upon TSA treatment. Other elements driven by a subtype I LTR giving rise to transcripts showed intermediate enrichment levels, whereas the analysed subclass III and IV elements, which were hardly expressed under these conditions, exhibited lowest levels of active chromatin marks. Nilsson and Bohm earlier proposed a model stating that the enhancer design of VL30 elements determines tissue specific expression and the response to external stimuli [50]. Our data support this model. Subtype I U3 promoters seem to be specifically susceptible to activation in this cell type. This could also be observed in arrested Swiss 3T3 fibroblasts. When treated with Anisomycin and TSA, 5 out of 14 (36%) transcripts were derived from an element with a subtype I U3 region; when left untreated we could only detect transcripts derived from subtype III elements (7 out of 7) (Table S1). Nevertheless, it remains unclear, which features distinguish the highly expressed hybrid element from other VL30 elements. Examination of the genomic environment did not reveal any obviously unusual architecture, the closest protein coding genes being located 764kb upstream and 16kb downstream of the element.

### VL30—an unusual type of retrotransposon

Our finding that VL30 elements could be reactivated with Aza-dC was in accordance with DNA methylation as the dominant silencing mechanism for TEs and supported by previous reports [39]. However, it could not account for earlier findings illustrating that a multitude of other stimuli without link to DNA methylation was able to trigger VL30 expression [27], [39], [51], [71]. Therefore, DNA methylation might be only one of several independent silencing pathways. Alternatively, Aza-dC treatment could be the cause for indirect effects leading to VL30 upregulation independent of DNA methylation levels at VL30 elements. Interestingly, loss of Dnmt1 in mouse brain tissue is not sufficient to influence VL30 expression (S.L. and C.S., unpublished data), although other stimuli have been reported to efficiently trigger VL30 in neurons [71]. This observation supports a model that Aza-dC might be an indirect trigger for VL30 activation.

Other mammalian TEs also respond to external signals, e.g. SINE elements can be reactivated *via* stress as well as Adenovirus infection [24], [25], LINEs by benzo(a) pyrene [26]. In cancer cells, TE silencing is impaired probably due to altered DNA methylation patterns [72], [73], and several human diseases are associated with a relaxation of TE repression [74]. Nonetheless, the regulation of VL30 expression is unusual among mammalian retrotransposons; a diverse repertoire of stimuli is able to highly induce transcription in several cell systems such as fibroblasts, keratinocytes, haematopoietic, and neuronal cells [27], [39], [51]. In contrast to many other TEs, VL30 elements seem to exist in a default state, which is “poised for transcription”, without stringent silencing mechanisms. External stimuli can easily flip the switch to “active transcription”. There are several feasible explanations for this exceptional situation: (1) it may mirror the unusual life cycle of VL30 elements. They can be packed into MuLV (murine leukaemia virus) viral particles and get horizontally transferred, rather than vertically propagated as endogenous parasites [27]. For such a life cycle it is essential to be efficiently transcribed in somatic cells. Of special benefit could be a system that allows the elements to “sense” the physiological situation of the host. Stressful conditions might trigger high-level transcription and increase the chance to escape the host successfully *via* virus-mediated horizontal exit strategies. (2) Song and colleagues recently showed that VL30 RNA can interact with the DNA-binding protein PSF and modulate PSF-mediated repression of target genes [75], [76]. Therefore, VL30 elements could have acquired physiological functions in murine cells and might be necessary for the regulation of certain host genes (a process, called molecular domestication [77]).

### VL30 expression is synergistically regulated by histone acetylation and phosphorylation

Another novel finding of our study is the positive impact of histone H3 phosphoacetylation on VL30 transcription. For several years, mechanistic links between histone acetylation and histone phosphorylation caused by different stimuli, including stress and growth factors, have been known. For example, immediate early genes such as c-myc, c-fos, c-jun, TFF1 [54]–[56], [78], but also others [57], depend on the induction of histone H3 phosphoacetylation.

In a series of experiments we could show that VL30 expression was synergistically activated by stimuli leading to histone phosphorylation (FCS, anisomycin) and histone acetylation (TSA); full transcriptional activity was correlated with the formation of phosphoacetylated histone proteins at the VL30 LTR. Inhibition of the nucleosomal response by H89 abolished VL30 phosphoacetylation and expression, indicating that the formation of this dual histone mark was necessary for activation caused by FCS/TSA or anisomycin/TSA and that MSK1/2 might be the responsible kinase responsible for phosphorylation. In summary, we collected data supporting a model that VL30 transposable elements are novel phosphoacetylation target genes. Still many details about the exact mechanism of VL30 regulation *via* dual histone modification are unclear, and it remains to be determined which are the responsible HAT(s), HDAC(s) and phosphatase(s) regulating dynamic acetylation and phosphorylation. In this context it will be interesting to investigate putative connections between enhancer design, transcription factor binding, and recruitment of histone modifiers dependent on the VL30 subtype.

### Transposition rates of several TEs are not influenced by HDAC inhibition

The link between TE transcription and transposition is fundamental. Production of an adequate amount of mRNA is an essential premise for all subsequent steps in the lifecycle of a TE. Having monitored the influence of HDAC inhibition on transcription of several TEs, we also examined, if there is an influence on subsequent events resulting in altered transposition activity. We used an experimental system, which allowed us to survey transposition rates of episomally delivered LTR and LINE elements upon HDAC inhibition. We detected only minor differences in the frequency of retrotransposition events when cells were treated with TSA, *i.e.* transposition events were slightly increased upon TSA treatment. We also observed a comparable enrichment of the transcript. Therefore, we could not detect obvious effects on transposon activity exceeding effects coherent with enhanced transcription. Final answers on the issue of histone- and protein-acetylation, influencing the process of transposition in mammals, will certainly demand further and more detailed investigations.

### Implications for HDAC inhibitor treatment of humans

HDAC inhibitors are promising anticancer drugs because they can change gene expression patterns in cancer cells, leading to cell cycle arrest or apoptosis [4]. More recently, their use for the treatment of non-cancer diseases has also been suggested [79], [80]. Our finding that HDAC inhibition can reactivate VL30 elements in mice might indicate that other TEs or non-coding elements could also respond to an epigenetic therapy in humans. Therefore, careful evaluation of potential effects beyond the deregulation of single copy genes will be an important issue for future studies. An interesting aspect – along with other dangers arising from increased TE activity – will be their emerging potential to modulate cellular regulatory networks. An exiting example for such a pathway conserved in mice and humans is the interaction of the PSF protein and VL30 RNA in mice and other non-coding TE-derived RNAs in human cells, respectively, affecting tumourigenesis [81].

## Materials and Methods

### Cell lines

Wildtype (HDAC1^+/+^) and corresponding HDAC1^−/−^ fibroblasts cell lines were established from E13.5 embryos carrying two floxed alleles of the Hdac1 gene [82]. Primary fibroblasts (mixed 129SvOla/Bl6, more than 80% being of Bl6 origin) were isolated and immortalised following the 3T3 immortalisation protocol [83]. Subclones were either transfected with a control vector or a vector encoding the cre recombinase leading to the deletion of exon 6 encoding the catalytic core of HDAC1. Cells were maintained in DMEM supplemented with 10% foetal calf serum (FCS) and gentamycin. For growth arrest of cells, Swiss 3T3 fibroblasts were serum starved for 72h in DMEM containing 0.2% FCS.

### Inhibitor treatments

The following inhibitors were used in this study: trichostatin A (TSA) (50 ng/ml [166 nM]; Wako Pure Chemical Industries) was applied for 3–24h; 5′Aza-2′-deoxycytidine (Aza-dC) (1µM) for 24h followed by additional incubation of cells for 24h without inhibitor; anisomycin (Aniso) (50 ng/ml [189 nM]; Sigma) for 1–6h, and H89 (10 µM; Alexis Biochemicals) for 1h 15min. H89 was added 15min prior to anisomycin.

### Transposition assay

Plasmids carrying a tagged IAP, MusD, L1.Md or L1.Hs retrotransposon (described in [48]) were transfected into logarithmically growing U2OS cells maintained in DMEM with 10% FCS and gentamycin. Cells were split 24h prior to the transfection procedure and 1×10^5^ cells were seeded on a 6-well plate. Transfection was performed with 1µg of plasmid DNA and Lipofectamine (Invitrogen) following the manufacturer's instructions. After 6h, the medium was replaced by DMEM supplemented with 10% FCS and gentamycin. If required, 66nM TSA was added for 24h after transfection. To monitor expression levels of the transfected constructs, cells were lysed 24h post transfection with TRIzol reagent (Invitrogen), RNA was isolated, DNase treated, reversely transcribed and subjected to quantitative RT-PCR as described above. The following primers were used for normalisation: GAPDH-for TCT TCT TTT GCG TCG CCA G, GAPDH-rev AGC CCC AGC CTT CTC CA and determination of episomal TE expression: Neo-for ATC AGA CAG CCG ATT GTC TG, Neo-rev CAG TTC CGC CCA TTC TCC G. For the analysis of transposition rates, cells were transferred to a 10cm dish 24h post transfection and cultured for 1 week, split and reseeded on 10cm dishes (5×10^5^ per dish). Selection for neo-positive cells was started 24h later by adding 500µg/ml G418 to the growth medium. After 2 weeks, cell clones were fixed with 70% EtOH, stained with 0.1% methylene blue/0.5M Na-acetate solution and counted.

### Western blot and dot blot analysis

Histone and total protein preparation for Western blot analysis was performed as previously described [57]. The following antibodies were used: HDAC1 polyclonal antibody (Millipore 06-720), HDAC2 3F3 monoclonal antibody (Millipore 05-814), HDAC3 (Abcam ab16047), DNMT1 (Abcam ab13537), β-actin (Sigma A5316), H3ac (Millipore 06-599), H4ac (Millipore 06-598), H3K4me3 (Millipore 07-473), H3S10pK14ac (Millipore 07-081 and a rabbit serum raised against the H3 peptide ARTKQTARKS(ph)TGGK(ac)APRKQL in collaboration with Eurogentec, Belgium) and c-H3 (Abcam ab1791). Densitometric quantification of Western blots was performed with the Imagequant software. For Dot blot analysis, 1 microliter of each peptide (0.2 mg/ml and 0.04 mg/ml in water) was spotted on a PVDF membrane, antibody incubation and detection was performed by standard Western blot methods.

### DNA methylation analysis

To quantify DNA methylation levels at defined DNA sequences, we employed the MS-SNuPE technique as described elsewhere [32]. Primers for PCR: bs-IAP-rc-for GTT TTA GTA TAG GGG AAT GTT AGG GTT; bs-IAP-rc-rev ACC AAA AAA AAC ACC ACA AAC CAA AAT CTT CTA C. Primers for the SnuPE reaction: rcS789 GTA GTT AAT TAA GGA GTG ATA; rcS628 AGA TTT TTG TGT TGG GAG T; rcS894 GAA GAT GTA AGA ATA AAG TTT TGT.

### Chromatin immunoprecipitation assay

Preparation of soluble chromatin and chromatin immunoprecipitation assays were carried out as described previously [57]. Antibodies for immunoprecipitation of modified histones were obtained from Millipore (H3ac 06-599, H4ac 06-598, H3S10p/H3K14ac 07-081) or generated in collaboration with Eurogentec, Belgium (H3S10p/H3K14ac), the antibody recognising C-terminal histone H3 was ordered from Abcam (c-H3 ab1791). H3K9me2 and H3K27me3 antibodies were a kind gift of T. Jenuwein, MPI Freiburg. As negative control unspecific IgGs from preimmunsera were used. Quantification of precipitated material was performed *via* quantitative Real-Time PCR employing an iCycler iQ system from Bio-Rad, SYBER-green (Molecular probes) and Taq DNA Polymerase (Fermentas) or *via* semi-quantitative PCR using a Biometra T3 thermocycler and the GoTaq PCR Master mix from Promega followed by visualisation of DNA fragments on an ethidium bromide stained 2% agarose-TAE gel. Primers have been described elsewhere [15], [84] or can be found in Table S2.

### Expression analyses

RNA was extracted with TRIzol Reagent (Invitrogen), possible genomic DNA contamination was removed using Turbo DNase (Ambion) and cDNA was synthesised with the iScript cDNA Synthesis Kit (Bio-Rad). For quantification of transcripts we performed RT-PCR on an iCycler iQ system from Bio-Rad using SYBER-green (Molecular probes) and Taq DNA Polymerase (Fermentas). Primers sequences have been described previously [15], [84] and are given in Table S2.

### Cloning of genomic and expressed transposable elements

To characterise TE transcripts, RNAs from three independent biological samples from untreated, TSA and Aza-dC treated fibroblasts were harvested, extracted with TRIzol Reagent (Invitrogen), DNase treated (Turbo DNase, Ambion), reversely transcribed (iScript, Bio-Rad) and used for PCR amplification in doublets (Biometra T3 thermocycler, GoTaq PCR Master mix from Promega; primers see Table S2). As control, PCR reactions were also performed with genomic DNA as template, amplifying genomic elements. The resulting six PCR reactions for each condition were pooled (to exclude a bias due to PCR reactions), loaded on an ethidium bromide stained agarose-TAE gel, eluted and purified using the Wizard Plus SV Gel and PCR Clean Up System (Promega). DNA fragments were cloned into the pGEM-T vector system (Promega) and transformed into E.coli (DH5α). Positive clones were grown in LB liquid medium, plasmid DNA was extracted using the Wizard Plus SV Miniprep DNA Purification System (Promega) and sequenced.

## Supporting Information

Figure S1 MuLV LTR element expression. mRNA was isolated from logarithmically growing wildtype fibroblasts (HDAC1^+/+^) or HDAC1 deficient fibroblasts (HDAC1^−/−^) and from wildtype fibroblasts treated with TSA (166nM; 24h) or Aza-dC (1µM; 24h, 24h recovery). Expression levels were determined with qRT-PCR. Values are normalised to HPRT expression and are shown relative to the expression in untreated HDAC1^+/+^ fibroblasts. N = 3; ± SD.
(0.12 MB TIF)

Figure S2 TSA treatment has only minor impact on the transposition frequency of retrotransposons. (A) Representation of a retroelement marked with a neo reporter gene. The retroelement carries its set of functional genes, allowing autonomous retrotransposition and a neo cassette which is placed in reverse orientation, interrupted by an intron framed by a splice donor (SD) and splice acceptor site (SA) in forward orientation. To activate the expression of the neo gene from its promoter (Pr), retrotransposition including the steps of transcription, splicing, reverse transcription and integration, has to occur. (B) Experimental procedure to detect retrotransposition rates and expression levels of the tagged element. After transfection, cells were treated with 66nM TSA for 24h to determine the effect of HDAC inhibition on transposition frequency determined by quantification of G418 resistant clones. To monitor the influence of HDAC inhibition on expression, mRNA abundance was measured via Real Time PCR. Primers were designed for the neo gene cassette and values were normalised against levels of the housekeeping gene GAPDH. (C) Analysis of the activity of murine LTR elements MusD and IAP, the murine non-LTR element L1.Md and the human non-LTR element L1.2B in U2OS cells upon TSA treatment. Transposition events were determined by counting G418 resistant clones fixed with EtOH and stained with methylene blue. The numbers of clones in TSA treated cells are given relative to the numbers of clones in untreated cells (Transposition rate). Expression of tagged retrotransposons was determined measuring the abundance of neo mRNA (Expression). Values for mRNA levels upon TSA treatment are given in percentage based on the mRNA levels determined in untreated cells. Values represent mean values of at least three independent experiments. ± SD.
(1.10 MB TIF)

Figure S3 Characterisation of transcribed IAP elements reveals preferential expression of defined IAP subtypes upon stimulation. (A) Schematic view of a full length IAP retrotransposon. Three partly overlapping open reading frames encoding the *gag*, *prt* and *pol* genes are flanked by long terminal repeats (LTRs). The LTRs are comprised of a U3 region, followed by the R and U5 region. Primers used to amplify fragments of genomic and transcribed IAP elements from untreated, TSA and Aza-dC treated cells are indicated as black arrows. (B) Partial alignment of IAP R regions shows that the CT-rich region consists of a variable number of tandem repeat sequence motifs. This TCTCTCTTGCTTC core motif contains a binding site for the transcription factor CCAAT/enhancer-binding protein alpha (C/EBPα). The selected sequences were derived from public databases and correspond to previously described IAP elements (M17751; AC012382; AB99818) or sequences obtained during the analysis of expressed IAP elements in fibroblasts (G 06; T 18; A 22). (C) The length of a highly polymorphic CT-rich stretch within the R region of IAP elements is increased in IAP elements expressed upon Aza-dC treatment. Number of R regions investigated per condition: genomic: N = 27; untreated: N = 26; TSA: N = 31; Aza-dC: N = 30.
(0.48 MB TIF)

Figure S4 Characterisation of the antibody directed against histone H3 concomitantly phosphorylated at serine 10 (H3S10p) and acetylated at lysine 14 (H3K14ac). 40 and 200 ng of each peptide corresponding to aa 1–20 of histone H3 unmodified or with the indicated modifications were spotted on a PVDF membrane and probed with the H3KS10ph14ac antiserum. In parallel, 1000 ng of each peptide from the same dilution series were spotted on a duplicate membrane and stained with Ponceau S.
(0.84 MB TIF)

Figure S5 H3 phosphoacetylation marks VL30 elements in 3T3 fibroblasts. H3S10phK14ac levels at defined genomic VL30 elements in logarithmically growing fibroblasts untreated or treated with TSA (12h; 166nM) was determined via ChIP as described in Figure 4C.
(0.28 MB TIF)

Table S1 Characterisation of cloned IAP and VL30 elements Sheet IAP: Characteristic features of cloned IAP elements. Clones G 01 to G 27 correspond to IAP elements amplified from genomic material, 0 01 to 0 26 to IAP elements expressed in untreated cells, T 01 to 31 and A 01 to 30 to IAPs expressed upon TSA or Aza-dC treatment, respectively. Given is the identity of the cloned sequence to the closest related genomic sequence (Identity), the corresponding genomic contig (Contig), the chromosomal coordinates (Chrom. Coordinates) on the mm9 genome assembly (July 2007), chromosome number (Chromosome), length of the genomic IAP element (bp), and affiliation to one of the three subgroups: full length, IΔ1 deletion type or IAP carrying another deletion (Type). Finally, the LTR is characterised in terms of length (bp), length of the CT-rich hypervariable region in the R region (CT bp) and identity of the 5′ LTR to the 3′LTR (Identity). The list is further extended by the characterisation of eight IAP elements published earlier (M17551, AB099818, AC012382, AC087891, AC090431, AL590616, X04120, AxinFu). Sheet VL30: Characteristic features of cloned VL30 elements. The first column indicates, the template for DNA amplification (G) genomic DNA, (0) cDNA from untreated fibroblasts, (T) cDNA from TSA treated fibroblasts, (A) cDNA from Aza-dC treated fibroblasts, (AT) cDNA from Anisomycin+TSA treated fibroblasts. Given is the identity of the cloned sequence to the most similar genomic sequence (Identity), the corresponding genomic contig (Contig), the chromosomal coordinates (Chrom. Coordinates) on the mm9 genome assembly (July 2007), chromosome number (Chromosome) and length of the genomic VL30 element (bp). The LTR is characterised in terms of length (bp), identity between 5′ LTR and 3′LTR (Identity) and the affiliation of the U3 region to one of the four described subgroups (I–IV).
(0.06 MB XLS)

Table S2 List of TE-specific primers used in this work. Given are primer sequences used for quantification of ChIP experiments, expression analyses and cloning of TE derived transcripts. The numbers underneath the DNA sequences indicate how many TE copies of each family are expected to be amplified with the primers chosen. *In silico* analyses for testing primer specificities and estimating TE copy numbers were performed via NCBI Primer-Blast (http://www.ncbi.nlm.nih.gov/tools/primer-blast/index.cgi?LINK_LOC=BlastHome) by searching candidate TE primer sets against the Mus musculus genome database (taxid: 10090) under high stringency primer conditions, i.e., allowing not more than one mismatch per primer. Only hits of expected fragment size were counted as individual TE copies along the mouse chromosomes.
(0.02 MB XLS)
