# Supplementary figures and images for: Epigenetic Regulation of a Murine Retrotransposon by a Dual Histone Modification Mark
Source: PLoS Genet. 2010 Apr 29;6(4):e1000927. doi: 10.1371/journal.pgen.1000927 (PMC2861705; doi:10.1371/journal.pgen.1000927)

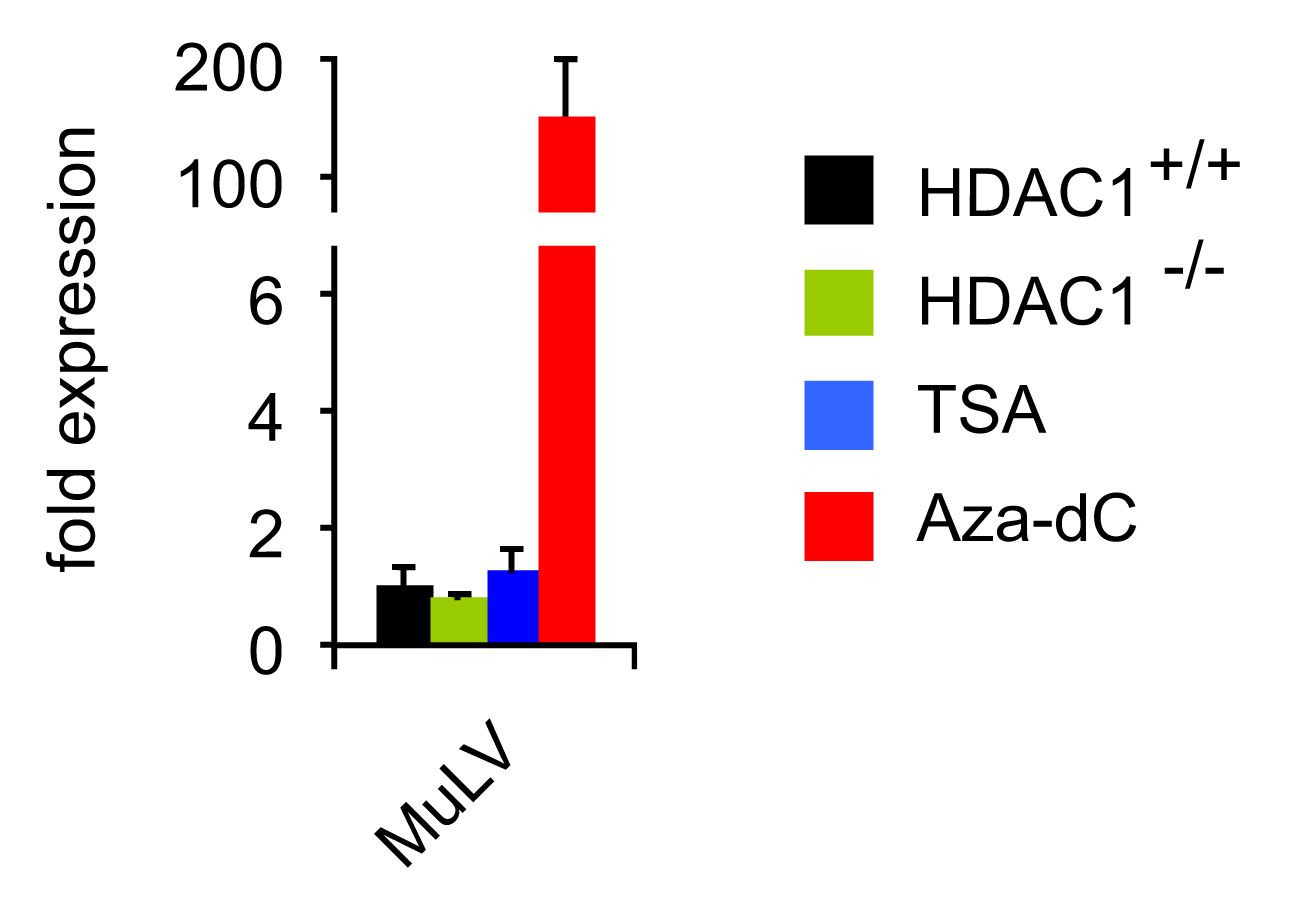

Supplement: Figure S1 — MuLV LTR element expression. mRNA was isolated from logarithmically growing wildtype fibroblasts (HDAC1+/+) or HDAC1 deficient fibroblasts (HDAC1−/−) and from wildtype fibroblasts treated with TSA (166nM; 24h) or Aza-dC (1µM; 24h, 24h recovery). Expression levels were determined with qRT-PCR. Values are normalised to HPRT expression and are shown relative to the expression in untreated HDAC1+/+ fibroblasts. N = 3; ± SD. (0.12 MB TIF) [file pgen.1000927.s001.tif]

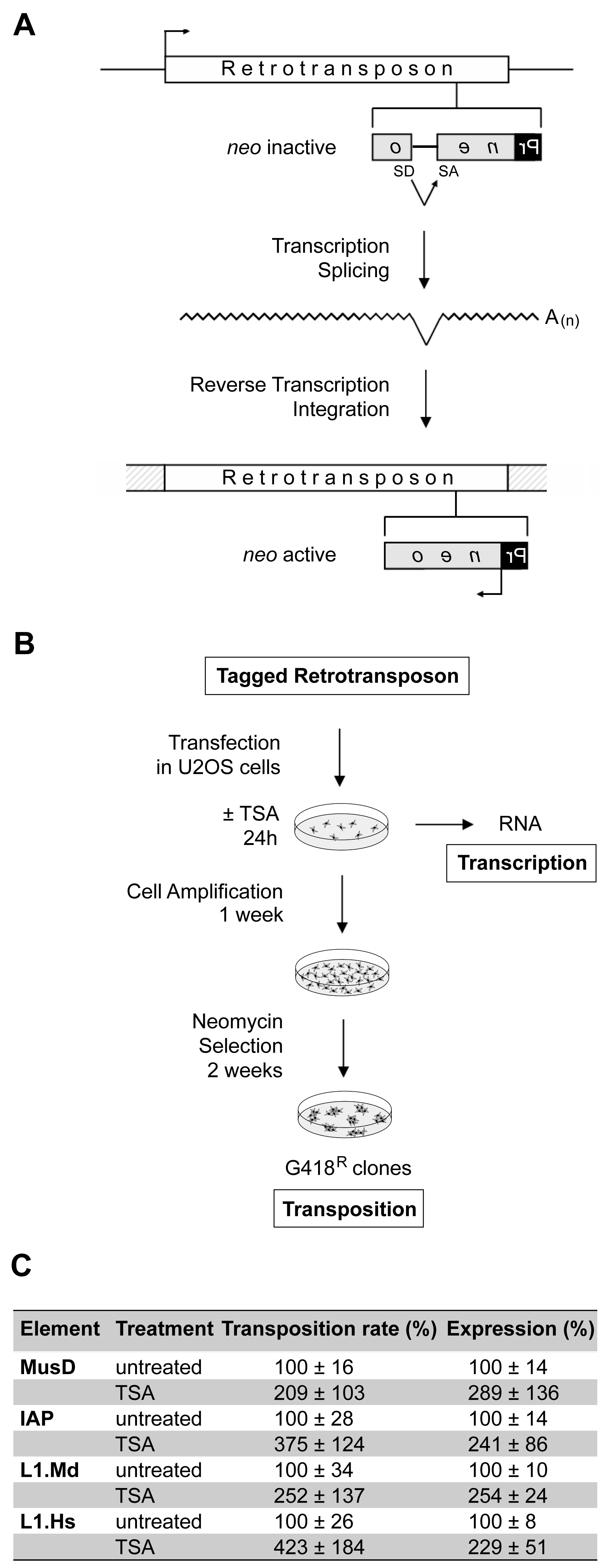

Supplement: Figure S2 — TSA treatment has only minor impact on the transposition frequency of retrotransposons. (A) Representation of a retroelement marked with a neo reporter gene. The retroelement carries its set of functional genes, allowing autonomous retrotransposition and a neo cassette which is placed in reverse orientation, interrupted by an intron framed by a splice donor (SD) and splice acceptor site (SA) in forward orientation. To activate the expression of the neo gene from its promoter (Pr), retrotransposition including the steps of transcription, splicing, reverse transcription and integration, has to occur. (B) Experimental procedure to detect retrotransposition rates and expression levels of the tagged element. After transfection, cells were treated with 66nM TSA for 24h to determine the effect of HDAC inhibition on transposition frequency determined by quantification of G418 resistant clones. To monitor the influence of HDAC inhibition on expression, mRNA abundance was measured via Real Time PCR. Primers were designed for the neo gene cassette and values were normalised against levels of the housekeeping gene GAPDH. (C) Analysis of the activity of murine LTR elements MusD and IAP, the murine non-LTR element L1.Md and the human non-LTR element L1.2B in U2OS cells upon TSA treatment. Transposition events were determined by counting G418 resistant clones fixed with EtOH and stained with methylene blue. The numbers of clones in TSA treated cells are given relative to the numbers of clones in untreated cells (Transposition rate). Expression of tagged retrotransposons was determined measuring the abundance of neo mRNA (Expression). Values for mRNA levels upon TSA treatment are given in percentage based on the mRNA levels determined in untreated cells. Values represent mean values of at least three independent experiments. ± SD. (1.10 MB TIF) [file pgen.1000927.s002.tif]

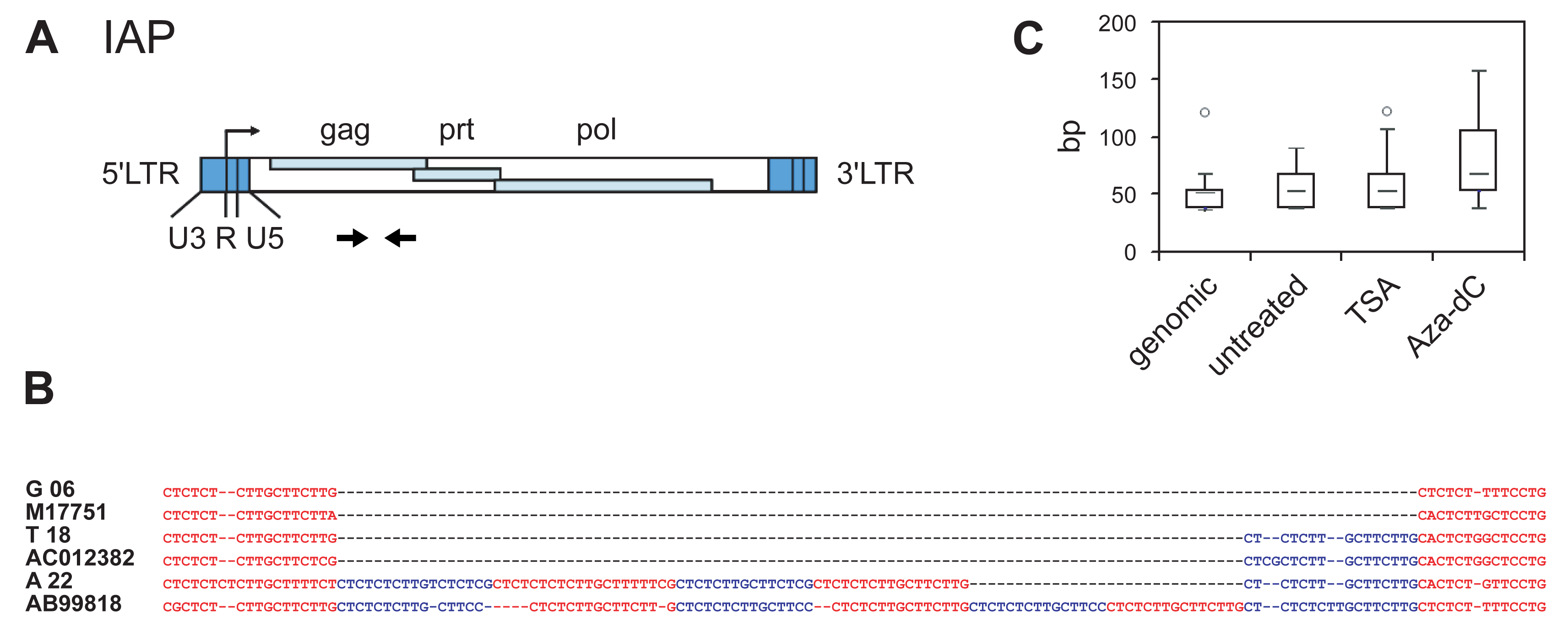

Supplement: Figure S3 — Characterisation of transcribed IAP elements reveals preferential expression of defined IAP subtypes upon stimulation. (A) Schematic view of a full length IAP retrotransposon. Three partly overlapping open reading frames encoding the gag, prt and pol genes are flanked by long terminal repeats (LTRs). The LTRs are comprised of a U3 region, followed by the R and U5 region. Primers used to amplify fragments of genomic and transcribed IAP elements from untreated, TSA and Aza-dC treated cells are indicated as black arrows. (B) Partial alignment of IAP R regions shows that the CT-rich region consists of a variable number of tandem repeat sequence motifs. This TCTCTCTTGCTTC core motif contains a binding site for the transcription factor CCAAT/enhancer-binding protein alpha (C/EBPα). The selected sequences were derived from public databases and correspond to previously described IAP elements (M17751; AC012382; AB99818) or sequences obtained during the analysis of expressed IAP elements in fibroblasts (G 06; T 18; A 22). (C) The length of a highly polymorphic CT-rich stretch within the R region of IAP elements is increased in IAP elements expressed upon Aza-dC treatment. Number of R regions investigated per condition: genomic: N = 27; untreated: N = 26; TSA: N = 31; Aza-dC: N = 30. (0.48 MB TIF) [file pgen.1000927.s003.tif]

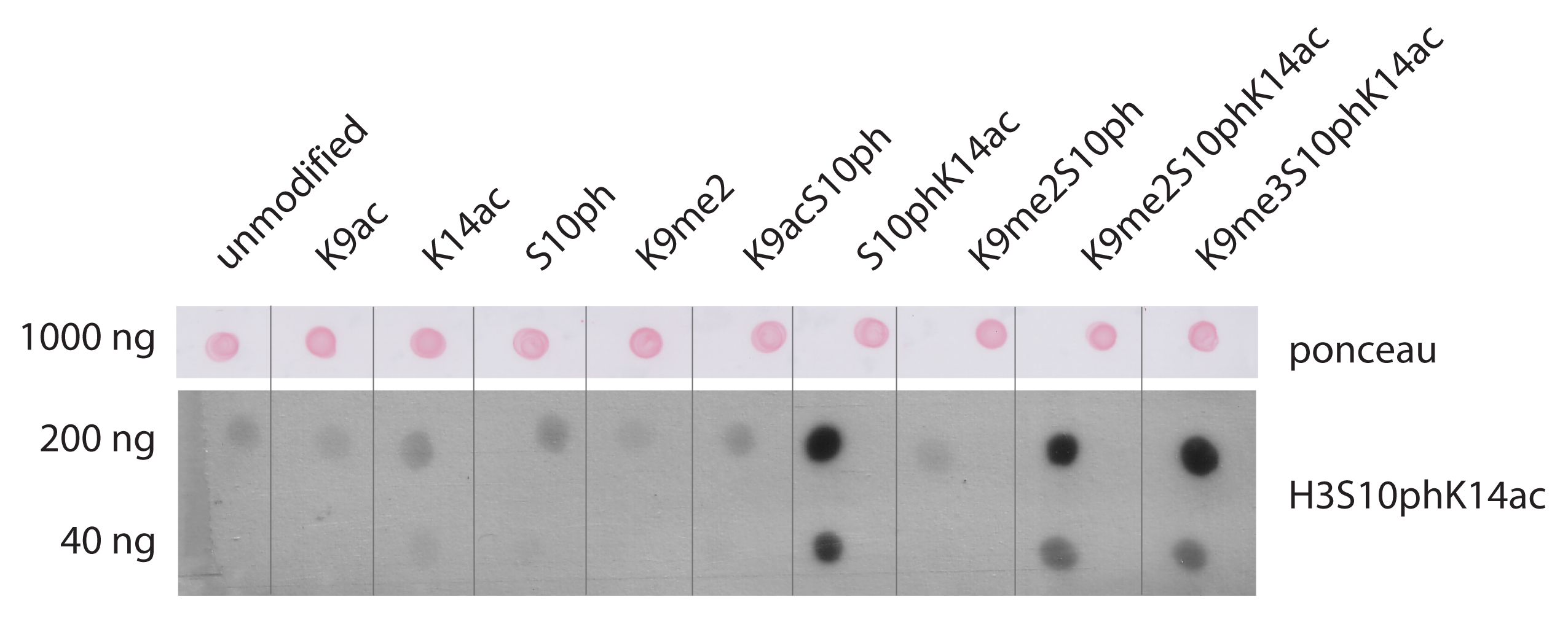

Supplement: Figure S4 — Characterisation of the antibody directed against histone H3 concomitantly phosphorylated at serine 10 (H3S10p) and acetylated at lysine 14 (H3K14ac). 40 and 200 ng of each peptide corresponding to aa 1–20 of histone H3 unmodified or with the indicated modifications were spotted on a PVDF membrane and probed with the H3KS10ph14ac antiserum. In parallel, 1000 ng of each peptide from the same dilution series were spotted on a duplicate membrane and stained with Ponceau S. (0.84 MB TIF) [file pgen.1000927.s004.tif]

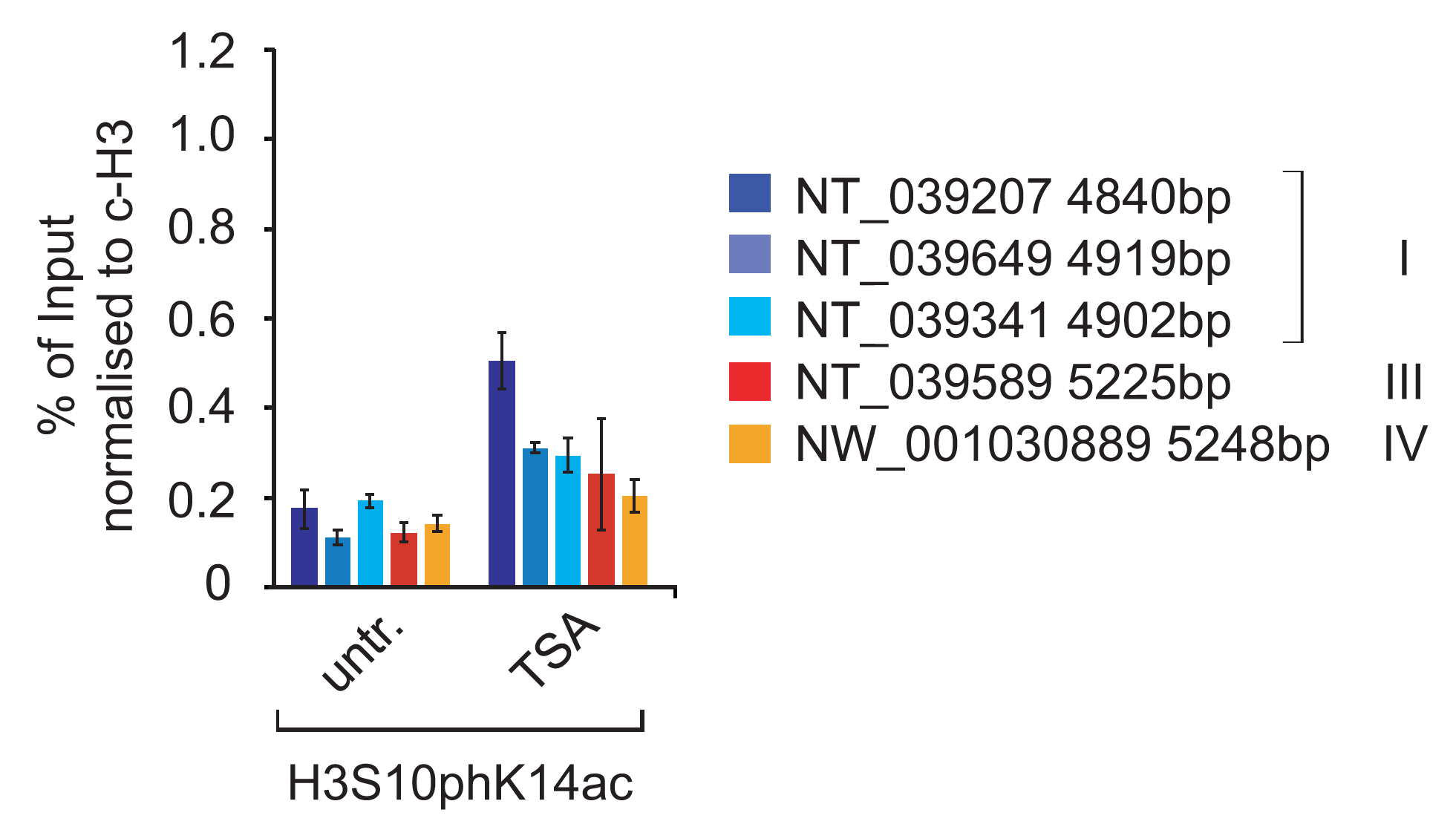

Supplement: Figure S5 — H3 phosphoacetylation marks VL30 elements in 3T3 fibroblasts. H3S10phK14ac levels at defined genomic VL30 elements in logarithmically growing fibroblasts untreated or treated with TSA (12h; 166nM) was determined via ChIP as described in Figure 4C. (0.28 MB TIF) [file pgen.1000927.s005.tif]
